# Linking Human Milk Oligosaccharides, Infant Fecal Community Types, and Later Risk To Require Antibiotics

**DOI:** 10.1128/mBio.03196-19

**Published:** 2020-03-17

**Authors:** Bernard Berger, Nadine Porta, Francis Foata, Dominik Grathwohl, Michèle Delley, Deborah Moine, Aline Charpagne, Léa Siegwald, Patrick Descombes, Philippe Alliet, Giuseppe Puccio, Philippe Steenhout, Annick Mercenier, Norbert Sprenger

**Affiliations:** a Department of Gastro-Intestinal Health, Nestlé Institute of Health Sciences, Nestlé Research, Société des Produits Nestlé SA, Lausanne, Switzerland; b Clinical Development Research Unit, Nestlé Research, Société des Produits Nestlé SA, Lausanne, Switzerland; c Department of Analytical Sciences, Institute of Food Safety & Analytical Sciences, Nestlé Research, Société des Produits Nestlé SA, Lausanne, Switzerland; d Jessa Hospital, Department of Paediatrics, Hasselt, Belgium; e Dipartimento Materno Infantile AOUP Paolo Giaccone, Università di Palermo, Palermo, Italy; f Nestlé Nutrition R&D, Vevey, Switzerland; Yale School of Public Health

**Keywords:** human milk oligosaccharides, 2′FL, LNnT, infant, formula, microbiota, *Bifidobacterium*, fecal community types, antibiotics

## Abstract

Human milk oligosaccharides (HMOs) may provide health benefits to infants partly by shaping the development of the early-life intestinal microbiota. In a randomized double-blinded controlled multicentric clinical trial, healthy term infants received either infant formula (control) or the same formula with two HMOs (2′-fucosyllactose and lacto-N-*neo*tetraose; test) from enrollment (0 to 14 days) to 6 months. Then, all infants received the same follow-up formula without HMOs until 12 months of age. Breastfed infants (BF) served as a reference group. Stool microbiota at 3 and 12 months, analyzed by 16S rRNA gene sequencing, clustered into seven fecal community types (FCTs) with marked differences in total microbial abundances. Three of the four 12-month FCTs were likely precursors of the adult enterotypes. At 3 months, microbiota composition in the test group (*n *= 58) appeared closer to that of BF (*n *= 35) than control (*n *= 63) by microbiota alpha (within group) and beta (between groups) diversity analyses and distribution of FCTs. While bifidobacteriaceae dominated two FCTs, its abundance was significantly higher in one (FCT BiH for *Bifidobacteriaceae* at high abundance) than in the other (FCT Bi for *Bifidobacteriaceae*). HMO supplementation increased the number of infants with FCT BiH (predominant in BF) at the expense of FCT Bi (predominant in control). We explored the association of the FCTs with reported morbidities and medication use up to 12 months. Formula-fed infants with FCT BiH at 3 months were significantly less likely to require antibiotics during the first year than those with FCT Bi. Previously reported lower rates of infection-related medication use with HMOs may therefore be linked to gut microbiota community types. (This study has been registered at ClinicalTrials.gov under registration number NCT01715246.)

## Abstract (precis)

Human milk is the sole and recommended nutrition for the newborn infant and contains one of the largest constituents of diverse oligosaccharides, dubbed human milk oligosaccharides (HMOs). Preclinical and clinical association studies indicate that HMOs have multiple physiological functions largely mediated through the establishment of the gut microbiome. Until recently, HMOs were not available to investigate their role in randomized controlled intervention trials. To our knowledge, this is the first report on the effects of 2 HMOs on establishing microbiota in newborn infants. We provide a detailed description of the microbiota changes observed upon feeding a formula with 2 HMOs in comparison to breastfed reference infants' microbiota. Then, we associate the microbiota to long-term health as assessed by prescribed antibiotic use.

## INTRODUCTION

At delivery, a microbiologically essentially sterile infant (1, 2) is exposed to a multitude of microbes from the mother and the environment (3, 4). The infant’s gut is progressively colonized with a dense microbial population. Donor effects are important, as seen from gut microbiota differences between Cesarean and vaginal deliveries (3, 5–8). However, nutrition also has an important impact on the composition of the gut microbiota, as seen from differences between breastfed and bottle-fed infants (8, 9) and from the cessation of breastfeeding (6). Neonatal gestational age (5, 10, 11), antibiotic therapy (12, 13), and diarrhea (14, 15) are additional factors affecting the development of the gut microbiome. Microbes play a key role in the development of the immune system (16–18) and host metabolism (19, 20). They are therefore speculated to exert a key impact on neonate and infant health that may last until later in life (21–23).

Milk is a rich biological fluid providing both protection and nutrition for the suckling newborns. Human milk contains nutrients and innate immune factors to support normal growth and development. Nondigestible and structurally diverse oligosaccharides, known collectively as human milk oligosaccharides (HMOs), form one of the major breastmilk components. They may support immune function through the modulation of the gut microbiome ecology, resulting in colonization resistance, and the establishment of an age-appropriate gut microbiota, educating the mucosal immune system in its development (16, 24, 25). Due to their structural similarity with mucosal glycans, HMOs may also function as soluble decoy receptors in the gut, protecting the neonate from enteric pathogens (26), and may directly interact with gut epithelial cells, yielding changes that may modulate host-microbial interactions (25).

In human milk, the oligosaccharides are extensions of lactose by one or more of the following monosaccharides: glucose, galactose, *N*-acetylglucosamine (GlcNAc), fucose, and sialic acid (*N*-acetylneuraminic acid) (25, 27). Three classes of oligosaccharides coexist: neutral fucosylated, neutral nonfucosylated with *N*-acetylglucosamine, and acidic with sialic acid. In contrast, cow’s milk contains very low levels of oligosaccharides, which are primarily neutral nonfucosylated with galactose only and acidic with sialic acid (27–29). Consequently, cow’s milk-based infant formula contains only relatively low levels of oligosaccharides, expected to be less than 100 mg/liter of the reconstituted formula (27), which, moreover, do not match with the major oligosaccharide classes found in human milk.

We previously reported on the primary outcome of a randomized double-blinded controlled multicentric safety clinical trial, in which a formula containing two major HMOs, namely, 2′-fucosyllactose (2′FL) and lacto-N-*neo*tetraose (LNnT), was found to be safe and well tolerated, allowing for age-appropriate growth of the infants (30). As part of the secondary objectives, we observed associations between feeding of the two-HMO formula and reduced rates for reported illnesses (in the lower respiratory tract) and infection-related medication use (antibiotics and antipyretics).

Here, we report on the impact of these HMOs (2′FL and LNnT) on the establishment of the gut microbiota, and we further explore its relationship with the reported illnesses and infection-related medication use.

## RESULTS

### Clinical trial.

**FIG 1:**
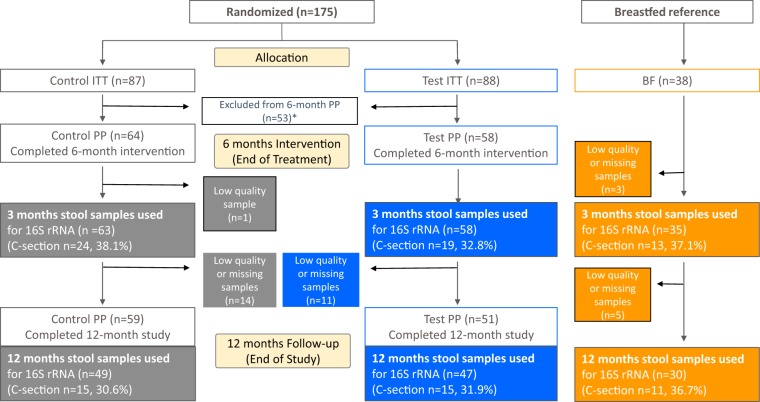
Flow of study participants. The number of stool samples analyzed at 3 and 12 months are specified for each arm and each delivery mode in the colored boxes. *, reasons for exclusion: 4 or more teaspoons (20 g) of complementary foods per day; being off study formula for 3 or more consecutive days before 4 months of age; disallowed medication use; out of visit window by more than 6 days; hospitalization for more than 3 consecutive days 1 week prior to visit date at 4 months visit. ITT, intention-to-treat; PP, per-protocol.

The randomized, double-blinded, controlled, multicenter interventional clinical trial with two parallel formula-fed groups was registered at ClinicalTrials.gov (registration number NCT01715246, 16 October 2012). Healthy term infants received either infant formula without HMOs (control group) or the same formula with two HMOs (1.0 g/liter 2′FL and 0.5 g/liter LNnT; test group) from enrollment to 6 months. Then, all infants received the same follow-up formula without HMOs until 12 months of age. A group of 38 infants exclusively breastfed (BF) since birth and whose mothers intended to exclusively breastfeed at least to 4 months was enrolled as a reference. From 4 months of age, complementary feeding (solid food) was allowed. The trial details and clinical findings related to the primary objective and supportive secondary objectives were recently published (30). The per-protocol (PP) infants who completed the 6-month treatment and for whom we had stool samples at 3 months of age represented 74% (control, 64/87) and 66% (test, 58/88) of the corresponding intention-to-treat population (Fig. 1). This well-controlled subpopulation was used to characterize the impact of the HMO supplementation on the stool microbiota.

### Taxonomic composition in the stool microbiota by 16S rRNA gene sequencing.

Stool samples were collected at 3 months and 12 months of age. Microbiota composition was determined by multiplexed high-throughput sequencing of amplicons obtained from the V3 and V4 regions of the 16S rRNA gene. After quality filtering, 16,014,421 sequences described the microbiota of 282 samples with an average coverage of 47,430 (median) sequences per sample classified into 336 operational taxonomic units (OTUs). Four samples of the per-protocol (PP) set with fewer than 10,000 sequences were excluded. Finally, the 3-month samples described 72% (control, 63/87) and 66% (test, 58/88) of the ITT population, and the 12-month samples described 56% (control, 49/87) and 53% (test, 47/88) of the ITT population (Fig. 1). Working with a subpopulation may affect the bias elimination of the study randomization. Therefore, we tested that the ITT population and the PP population reported here showed no bias in the baseline characteristics between the formula groups (Table 1) and no major difference in the clinical data (Table 2). Noteworthy, we used an approach allowing accurate annotation of the 16S rRNA sequences belonging to the genera *Bifidobacterium* (the dominant taxon at 3 months) and *Lactobacillus* down to the species or subspecies level (15). The taxonomic composition of all samples is reported at genus level in Fig. S1 in the supplemental material.

**TABLE 1:** Baseline characteristics of study participants

Infant characteristic	Value for:				
	ITT control (*n* = 87)^*a*^	ITT test (*n* = 88)^*a*^	PP control (*n* = 63)^*b*^	PP test (*n* = 58)^*b*^	BF reference (*n* = 35)^*b*^
Age (days)^*c*^	7.7 ± 3.3	8.6 ± 3.3	8.2 ± 3.2	8.7 ± 3.2	NA^*d*^
Male sex (*n*, [%])	44 (50.6)	44 (50.0)	33 (52.4)	29 (50.0)	25 (71.4)
Gestational age (wks)^*c*^	39.2 ± 1.0	39.2 ± 1.1	39.3 ± 1.1	39.2 ± 1.0	39.3 ± 1.1
Siblings at birth (*n* [% yes])^*e*^	58 (66.7)	56 (63.6)	41 (65.1)	35 (60.3)	20 (57.1)
Cesarean delivery (*n* [%])^*e*^	32 (36.8)	32 (36.4)	24 (38.1)	19 (32.8)	13 (37.1)
Wt (kg)^*c*^	3.4 ± 0.4	3.4 ± 0.4	3.3 ± 0.4	3.4 ± 0.4	3.4 ± 0.3
Length (cm)^*c*^	50.9 ± 1.9	50.7 ± 1.7	49.9 ± 1.8	50.1 ± 1.8	50.3 ± 1.5

a Some parameters were previously reported (30).

b Stool samples with good-quality 16S gene amplification coverage.

c Values are means ± standard deviations.

d NA, not applicable.

e No difference between PP control and PP test by two-tailed chi-square with Fisher’s exact probability test.

**TABLE 2:** Formula intake and adverse event and medication reporting

Reported event	OR (*P* value)^*a*^	No. (%) of infants or value			
		ITT control (*n* = 87)^*b*^	ITT test (*n* = 88)^*b*^	PP control (*n* = 63)^*c*^	PP test (*n* = 58)^*c*^
Formula intake (3 mo) (ml/day)^*d*^		898 ± 190	887 ± 182	908 ± 189	870 ± 179
Antibiotic use					
0–6 mo	0.6 (0.2)	43 (49.4)	30 (34.1)	32 (50.8)	22 (37.9)
0–12 mo	0.5 (0.07)	53 (60.9)	37 (42.0)	42 (66.7)	29 (50.0)
Antipyretic use					
0–6 mo	0.6 (0.18)	31 (35.6)	23 (26.1)	26 (41.3)	17 (29.3)
0–12 mo	0.7 (0.35)	35 (40.2)	28 (31.8)	30 (47.6)	22 (37.9)
Bronchitis					
0–6 mo	0.2 (0.004)	19 (21.8)	6 (6.8)	17 (27.0)	4 (6.9)
0–12 mo	0.3 (0.005)	24 (27.6)	9 (10.2)	22 (34.9)	7 (12.1)
LRTI (AE cluster)^*e*^					
0–6 mo	0.5 (0.19)	21 (24.1)	13 (14.8)	18 (28.6)	10 (17.2)
0–12 mo	0.5 (0.054)	30 (34.5)	17 (19.3)	26 (41.3)	14 (24.1)

a OD, odds ratio and result of Fisher’s exact test between PP control and PP test.

b Some parameters were previously reported (30).

c Stool samples with good-quality 16S gene amplification coverage.

d Means ± standard deviations.

e LRTI, lower respiratory tract infection; AE, adverse event.

10.1128/mBio.03196-19.1 FIG S1 Bubble plots of the 26 most dominant bacterial genera in stool samples at 3 months (3m) and 12 months (12m) of age. Each column describes one sample. The size of the squares depicts the relative abundance (bottom left code). Taxa are shown for each row at the right with family-level color-coded boxes. BF, breastfed; C, C-section delivered; V, vaginally delivered; *n*, number of samples. Download FIG S1, PDF file, 0.5 MB. Copyright © 2020 Berger et al. 2020 Berger et al. This content is distributed under the terms of the Creative Commons Attribution 4.0 International license.

### At 3 months, formula with two HMOs shifted stool microbiota composition and diversity toward that of BF infants.

**FIG 2:**
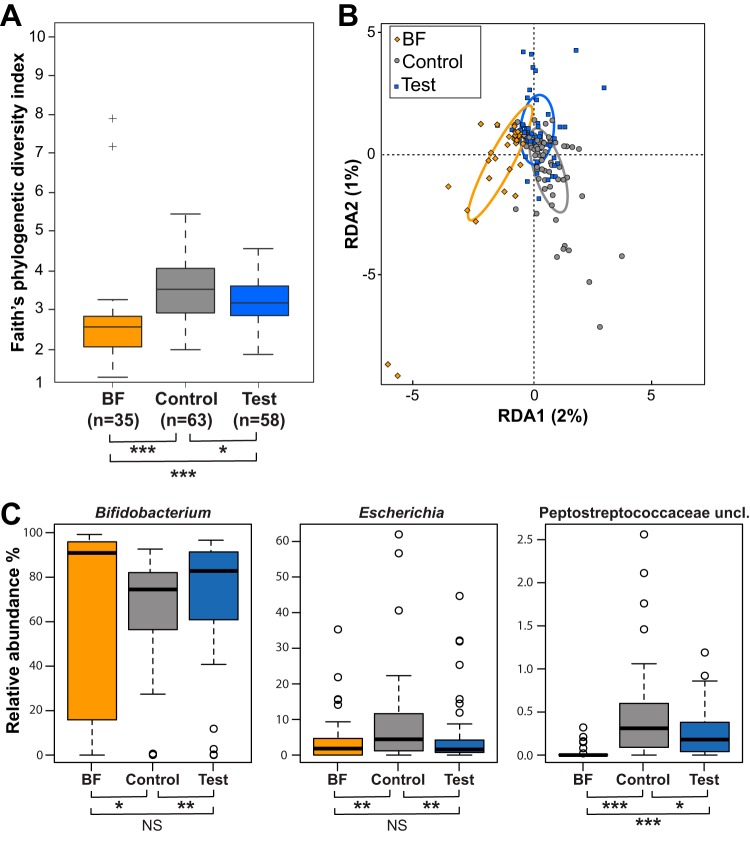
Comparison of microbiota compositions between feeding groups at 3 months of age. (A) Alpha diversity. Box plots of the phylogenetic distances measured by the Faith*’*s diversity index. (B) RDA at the genus level. Colored ellipses identify the 95% confidence interval around the centroid of each group. (C) Box plots of relative abundance for three genera at 3 months. Median values are shown in boxes encompassing the interquartile range; whiskers show the 5% to 95% range; crosses (diversity) or circles (genera) show the outliers. Significance by *t* test (diversity) or Mann-Whitney U test (genera): *, *P* < 0.05; **, *P* < 0.01; ***, *P* < 0.005; NS, not significant; BF, breastfed reference group. Sample size for each group is shown in panel A; *n*, number of samples.

**FIG 3:**
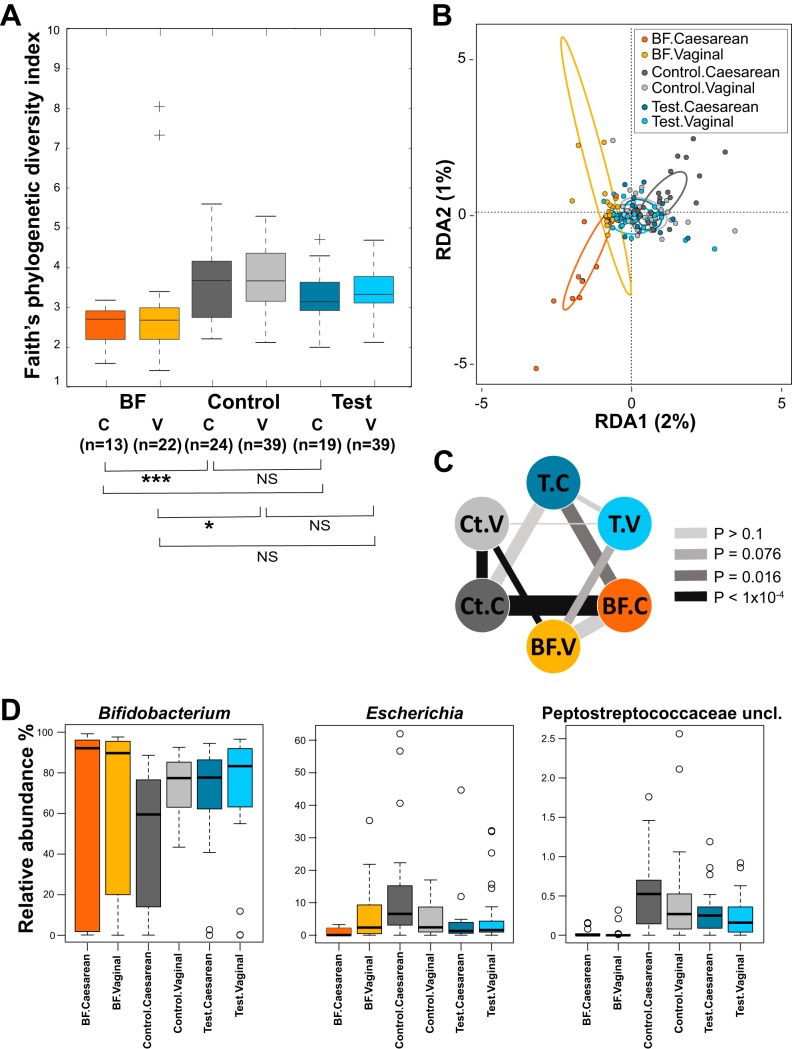
Comparison of microbiota compositions between feeding groups stratified by delivery modes at 3 months of age. (A) Alpha diversity. Box plots of the phylogenetic distances measured by the Faith’s diversity index. (B) RDA at the genus level. Colored ellipses identify the 95% confidence interval around the centroid of each group. Two outliers of the BF.vaginal group are not shown on this figure. (C) Dissimilarities of microbiota. The thickness of the lines represents the dissimilarity between groups (line weight is proportional to average pairwise Bray-Curtis distances at the genus level). Separation between groups was assessed by the distribution of pairwise distances (when between groups’ distances > within groups’ distances; assessed by Wilcoxon rank test) and shown as color-coded *P* values (the darker the color, the more significant the separation). (D) Box plots of relative abundances for three genera. Median values are shown in boxes encompassing the interquartile range; whiskers show the 5% to 95% range; crosses (diversity) or circles (genera) show the outliers. Significance by *t* test: *, *P* < 0.05; ***, *P* < 0.005; NS, not significant; BF, breastfed reference group; Ct, control group; T, test group; C, Caesarean delivery; V, vaginal delivery. Sample size for each group is shown in panel A; *n*, number of samples.

When comparing the phylogenic diversity (31) between feeding groups at 3 months, the lowest was in the BF group, and the test group was significantly lower than the control group (*P* < 0.05) and therefore closer to the BF group (Fig. 2A). Stratification by delivery mode showed a similar trend (Fig. 3A).

The global difference in microbiota compositions between feeding groups was statistically assessed by random permutations of redundancy analysis (RDA). At 3 months, the three groups were significantly separated at the genus level (*P* < 0.001) (Fig. 2B), with the test group closer to the BF group. Likewise, the test and control groups were significantly separated at the genus level (RDA1 component = 1%; *P* = 0.036). After stratification by mode of delivery, RDA at the genus level revealed a stronger contribution of the Caesarean-delivered infants to the overall difference between the formula-fed groups and the BF group (Fig. 3B). We calculated the Bray-Curtis distances between samples at the genus level and evaluated the separation between the test (T), the BF, and the control (Ct) groups (Fig. 3C and Table S1). As shown on Fig. 3C, the groups of Caesarean (C)-section delivered infants (the triangle T.C↔BF.C↔Ct.C) were more distantly related to each other than the groups of vaginally delivered infants (the triangle T.V↔BF.V↔Ct.V). Although the BF infants were always separated from the formula-fed infants, irrespective of delivery mode, the separation was clearer with the control group (Ct.C↔BF.C compared to T.C↔BF.C, and Ct.V↔BF.V compared to T.V↔BF.V). In the vaginally delivered infants, the separation between the test and BF groups did not reach significance (T.V↔BF.V). Noteworthy, in the test group, we could not distinguish between the delivery modes (T.C↔T.V), similarly to the BF group (BF.V↔BF.C). However, in the latter group, the average distances and variances were higher, probably due to the absence of standardization of the breast milk. On the contrary, the separation was very clear between delivery modes in the control group (Ct.V↔Ct.C). This stratified analysis of distances showed that the effect of the HMO supplementation was more pronounced in the population of Caesarean-delivered infants.

10.1128/mBio.03196-19.5 TABLE S1 Separation between feeding groups and delivery modes at 3 months assessed by the distribution of Bray-Curtis distances at the genus level. Download Table S1, PDF file, 0.6 MB. Copyright © 2020 Berger et al. 2020 Berger et al. This content is distributed under the terms of the Creative Commons Attribution 4.0 International license.

At 3 months, the abundances of the genera *Escherichia*, *Bifidobacterium*, unclassified *Peptostreptococcaceae*, and *Streptococcus* were modulated by the HMO supplementation, placing the test group closer to the BF group (Fig. 2C and Table S2). Regarding the impact of the delivery mode on the treatment effect for these genera (Fig. 3D), the control vaginally delivered (control.vaginal) and test Caesarean-delivered (test.Caesarean) groups were at similar levels, between the more contrasted control Caesarean-delivered (control.Caesarean) and test vaginally delivered (test.vaginal) groups. The HMO supplementation moved the microbiota profile seen in the C-section-born infants toward the profile observed in the vaginally delivered infants in the control group.

10.1128/mBio.03196-19.6 TABLE S2 Differences of taxa abundances at the genus level between control and test groups at 3 months. Download Table S2, PDF file, 0.6 MB. Copyright © 2020 Berger et al. 2020 Berger et al. This content is distributed under the terms of the Creative Commons Attribution 4.0 International license.

Since we used an approach allowing us to accurately annotate the 16S rRNA gene sequences belonging to the genus *Bifidobacterium* down to the species or subspecies level (15), we were able to establish that the HMO supplementation did not significantly change the relative abundance of the individual bifidobacterial species and subspecies, neither did the breastfeeding (see Table S3). In our data set, only 5% of the bifidobacteria belonged to B. longum subsp. *infantis*, the majority of bifidobacteria were identified as B. longum subsp. *longum* (18%), *B. breve* (15%), B. bifidum (12%), or *B. catenulatum* group (11%).

10.1128/mBio.03196-19.7 TABLE S3 Differences of abundances for bifidobacterial species between BF, control, and test groups at 3 months. Download Table S3, PDF file, 0.6 MB. Copyright © 2020 Berger et al. 2020 Berger et al. This content is distributed under the terms of the Creative Commons Attribution 4.0 International license.

### At 12 months (6 months after cessation of the intervention), no significant difference in microbiota composition was observed between the two formula groups.

Compared to that in the 3-month samples, the phylogenetic diversity measured at 12 months showed a clear increase in all feeding groups, although solely the difference between the control and BF groups remained significant at 12 months (see Fig. S2).

10.1128/mBio.03196-19.2 FIG S2 Comparison of alpha diversity between feeding groups at 3 months (3m) and 12 months (12m). Box plots of the phylogenetic distances measured by the Faith’s diversity index (*n*, number of samples). Median values are shown in boxes encompassing the interquartile range; whiskers show the 5% to 95% range; crosses show the outliers. Significance by *t* test: *, *P* < 0.05; ***, *P* < 0.005; NS, not significant; BF, breastfed reference group; *n* = number of samples. Download FIG S2, PDF file, 0.1 MB. Copyright © 2020 Berger et al. 2020 Berger et al. This content is distributed under the terms of the Creative Commons Attribution 4.0 International license.

At 12 months of age, the global microbiota analysis by RDA showed a significant ordination of the three groups at the genus level (RDA1 component = 2%; RDA2 component = 1%; *P* = 0.007). However, the two formula groups were not significantly separated anymore (RDA1 component = 1%; *P* > 0.1). Similarly, we found no significant differences in genus abundances between the two formula groups. Therefore, the effect of the HMO supplementation on the microbiota composition was not observed 6 months after cessation of the intervention.

### Seven fecal community types were defined in the 16S rRNA gene data set.

**FIG 4:**
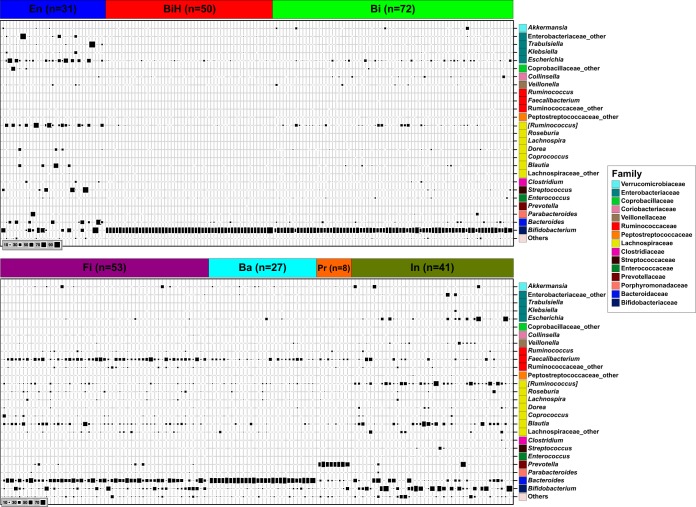
Bubble plot of the 26 most dominant bacterial genera in stool samples (at 3 and 12 months of age) clustered in seven FCTs. Each column describes one sample. The size of the squares depicts the relative abundance (bottom left code). Taxa are shown for each row at the right with family level color-coded boxes. *n*, number of samples.

Using a Dirichlet multinomial mixtures (DMM) modeling framework (32), we clustered the samples based on their profiles at the genus level. The optimal number of clusters to describe our data set (see Fig. S3) defined seven fecal community types (FCTs) showing very contrasted taxonomic compositions (Fig. 4) that were named with an abbreviation of their respective dominant taxa: FCT En for *Enterobacteriaceae*, FCT Bi for *Bifidobacteriaceae*, FCT BiH for *Bifidobacteriaceae* at higher abundance, FCT Fi for *Firmicutes*, FCT Ba for *Bacteroidaceae*, FCT Pr for *Prevotellaceae*, and FCT In for an intermediate state between 3- and 12-month FCTs (see details below).

10.1128/mBio.03196-19.3 FIG S3 DMM model fit is evaluated using the Laplace approximation to the model evidence for various values of K (the number of Dirichlet components). For this dataset, K = 7 results in the best fit. Download FIG S3, PDF file, 0.1 MB. Copyright © 2020 Berger et al. 2020 Berger et al. This content is distributed under the terms of the Creative Commons Attribution 4.0 International license.

**FIG 5:**
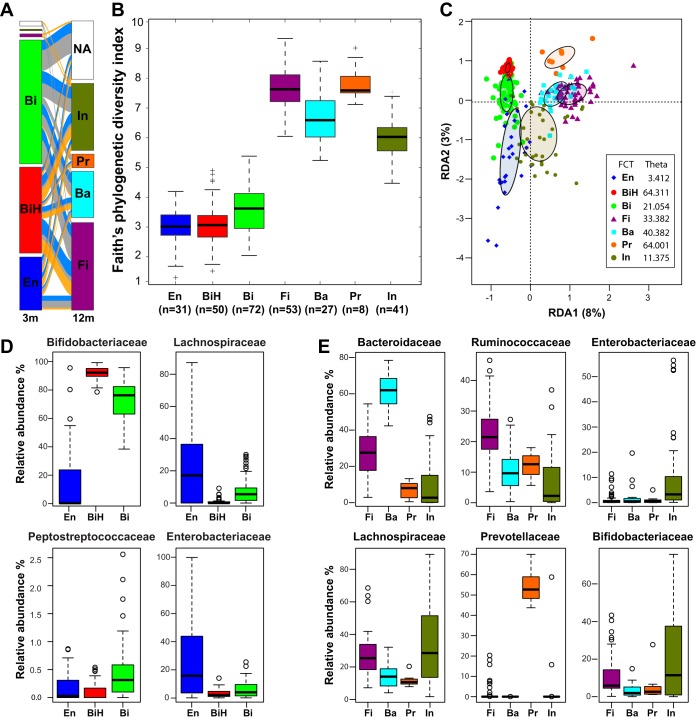
Description of the fecal community types. (A) Distribution of the FCTs at 3 and 12 months (3m and 12m, respectively). Transitions between the FCTs are depicted by the streams colored by feeding group (orange, BF; gray, control; blue, test). NA, sample not available. (B) Alpha diversity of the FCTs. Box plots of the phylogenetic distances measured by the Faith’s diversity index. Median values are shown as boxes encompassing the interquartile range; whiskers show the 5% to 95% range; crosses show the outliers. All FCTs showed a different distribution (*t* test; *P* value < 0.05) except for two comparisons: FCT En versus FCT BiH and FCT Fi versus FCT Pr. (C) RDA at the genus level. Filled ellipses indicate the 95% confidence interval (CI) around the centroids of each data set. (D) Box plots of relative abundances at the family level for dominant FCTs at 3 months. (E) Box plots of relative abundances at the family level for dominant FCTs at 12 months. Median values are shown as boxes encompassing the interquartile range; whiskers show the 5% to 95% range; circles show the outliers. Sample size for each group is shown in panel B; *n*, number of samples.

The homogeneity of the FCTs ranged from the less variable FCT BiH and FCT Pr, the latter also being the less frequent, to the most variable FCT En (theta values in Fig. 5C). Considering that the main differences of genus abundances between FCTs were rooted at the family level, we discuss them at the family level.

### At 3 months, the HMO supplementation decreased the number of infants with FCT Bi in favor of the BF-specific FCT BiH.

**FIG 6:**
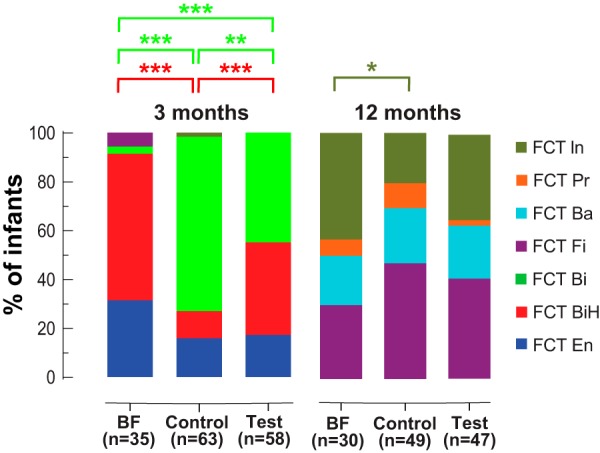
Distribution of fecal community types among feeding groups at two time points. Significant differences are shown using the FCT color code. *, *P* < 0.05; **, *P* < 0.01; ***, *P* < 0.001; NS, not significant; *n*, number of samples.

Samples from 3-month-old infants mostly harbored FCT En, FCT BiH, or FCT Bi (Fig. 4A). The most important differences between these FCTs were observed in *Bifidobacteriaceae*, *Lachnospiraceae*, *Peptostreptococcaceae*, and *Enterobacteriaceae* (Fig. 5D). Both FCT BiH and FCT Bi showed a high proportion of *Bifidobacteriaceae*, which was, however, higher in FCT BiH. FCT En was characterized by high levels of *Enterobacteriaceae* and *Lachnospiraceae* (Fig. 5C). FCT Bi, which showed an intermediate level of *Lachnospiraceae*, also harbored the highest content of *Peptostreptococcaceae*. The average phylogenetic diversity of these three FCTs showed FCT En = FCT BiH < FCT Bi (Fig. 5B). The distribution of the FCT BiH and FCT Bi at 3 months was associated with the feeding groups (Fig. 6). Supplementation of the formula with two HMOs increased the number of infants with FCT BiH (predominant in BF) at the expense of FCT Bi (predominant in control).

### At 12 months (6 months after cessation of the intervention), no significant association was detected between feeding groups and the four observed FCTs.

Between the four FCTs essentially observed at 12 months of age (Fig. 5A), the differences were mainly found in 6 families (Fig. 5E). FCT Fi, which showed the highest phylogenetic diversity among the FCTs (Fig. 5B), was also characterized by the highest proportion of *Ruminococcaceae* (mainly *Faecalibacterium*), with substantial contributions of *Bacteroidaceae* and *Lachnospiraceae*. FCT Ba was essentially driven by *Bacteroidaceae*. FCT Pr was the most homogeneous cluster at 12 months (Fig. 5C), with a clear dominance of *Prevotellaceae*. FCT In was the most heterogeneous cluster at 12 months (Fig. 5C), with the lowest phylogenetic diversity (Fig. 5B), the highest proportion of *Bifidobacteriaceae*, and remarkable contributions of two other dominant taxa of the 3-month communities (*Enterobacteriaceae* and *Lachnospiraceae*). Although samples from both FCT Fi and FCT In showed similar levels of *Lachnospiraceae*, the members of this family were more diverse in FCT Fi (with the highest proportion of *Faecalibacterium*), whereas FCT In contained mainly *Ruminococcus* and *Blautia* (Fig. 4). In RDA, FCT In appeared as an intermediate state between the 3-month FCTs and the other 12-month FCTs, with FCT Pr clearly separated from the others (Fig. 5C) (*P* < 0.001).

Although the frequency of FCT Fi and FCT In positions the test group as intermediate between the BF and control groups at 12 months of age, we did not observe a significant association between the formula types and the FCT distribution (Fig. 6). Stratification by delivery mode showed that the slight increase of FCT In in the test group was essentially driven by the Caesarean-delivered infants (57% compared to 25% among the vaginally delivered infants).

When we investigated the microbiota progression in each infant, the 3-month FCTs were not predictive of the 12-month FCTs (Fig. 5A).

### Modest differences of species relative abundances within the bifidobacterial population between FCT BiH and FCT Bi.

Although the FCTs were defined at the genus level, we investigated if the *Bifidobacterium* species distribution could underlie the difference in total bifidobacterial abundance observed between FCT BiH and FCT Bi. To control for the potential impact of other components of breast milk, we focused our analysis on the formula groups. Relative to the bifidobacterial population, differences of proportion between the FCT BiH and FCT Bi were only observed for Bifidobacterium adolescentis (means: 4% versus 3% of bifidobacteria, respectively; *P* = 0.03, false-discovery rate [FDR] = 0.15) and the B. catenulatum group (*B. catenulatum*, *B. pseudocatenulatum*, *B. angulatum*, *B. catenulatum*, and *B. catenulatum* subsp. *kashiwanohense*; means: 26% versus 14% of bifidobacteria, respectively; *P* = 0.04; FDR = 0.15). Notably, B. longum subsp. *infantis* was not differently abundant between FCT BiH and FCT Bi (means: 9% versus 4% of bifidobacteria, respectively; *P* = 0.16; FDR = 0.29).

### Difference of total bacterial loads between FCTs but not between feeding groups.

**FIG 7:**
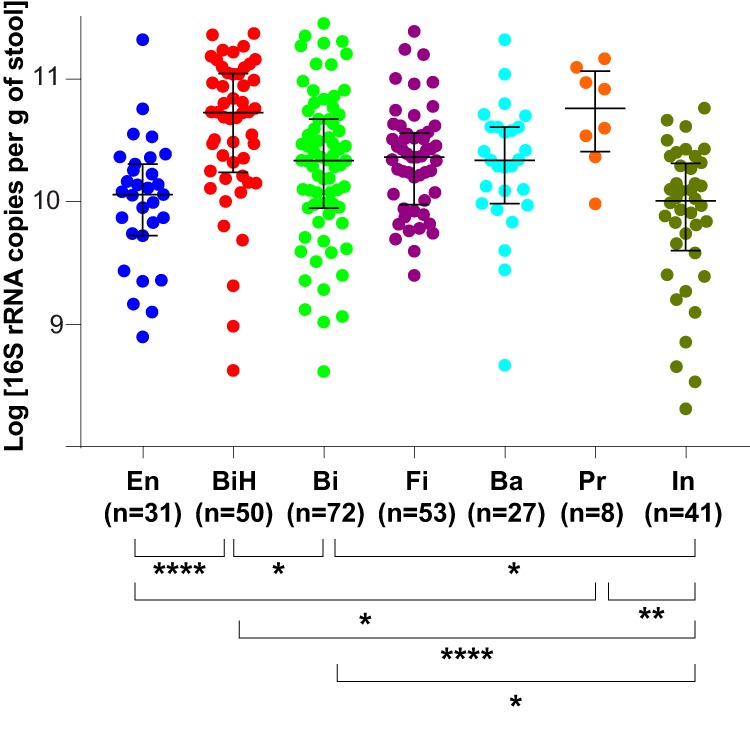
Comparison of total bacterial 16S rRNA copy numbers between FCTs. Medians with interquartile ranges. Only significant differences are indicated: *, *P* < 0.05; **, *P* < 0.01; ****, *P* < 0.0001; *n*, number of samples.

Since 16S rRNA sequencing only indicates relative proportions of stool bacteria, we complemented this information by measuring by quantitative PCR (qPCR) the total bacterial 16S rRNA gene content per gram of feces. No difference was observed between samples from the three feeding groups at either 3 or 12 months of age. In contrast, significant differences were observed between FCTs (Fig. 7), with FCT BiH and FCT Pr showing a higher 16S rRNA gene content, and FCT En and FCT In showing a lower one. Moreover, the differences in 16S rRNA contents may underestimate the difference of the bacterial count per gram of feces between FCTs, due to dominant taxa harboring very different 16S rRNA copy numbers (33; database at https://rrndb.umms.med.umich.edu/). For example, FCT BiH was mainly composed of *Bifidobacterium* harboring 3.4 (mean) copies per chromosome, whereas *Enterobacteriaceae* and *Lachnospiraceae*, harboring 6.9 copies and 5.9 copies, respectively, dominated the FCT En. Therefore, the difference of bacterial counts per gram of feces between these two FCTs is likely even more pronounced. Similar corrections increasing the difference of bacterial counts apply when comparing FCT Pr dominated by *Prevotellaceae* (3.8 copies) with the *Bacteroidaceae*-dominated (5.6 copies) FCT Ba.

### Association of the fecal community types with clinical parameters.

We further explored whether the FCTs at 3 months were predictive of the reported infection-related illnesses and medications until 12 months of age (30). We restricted the analysis to the randomized formula-fed population and tested 42 associations with reported clinical parameters (see Materials and Methods).

**FIG 8:**
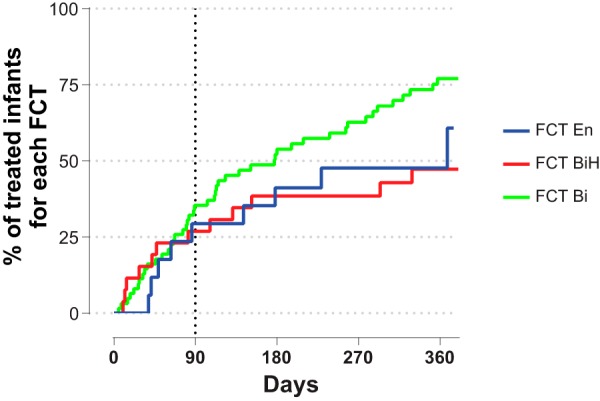
Associations of FCTs with antibiotics use. Kaplan-Meier plot of time to first antibiotics use by FCT (FCT En, FCT BiH, and FCT Bi); BF infants were not included in this analysis.

FCTs at 3 months associated prospectively with any antibiotic treatment up to 12 months. Infants with FCT BiH microbiota at 3 months were less likely to require antibiotics up to 12 months (odds ratio [OR], 0.4; 95% confidence interval [CI], 0.17 to 0.93; *P* = 0.033) than infants with FCT En or FCT Bi microbiota. On the other hand, infants with FCT Bi microbiota were more likely to require antibiotics during the first 12 months of life (OR, 3.3; 95% CI, 1.54 to 7.02; *P* = 0.0025) than those with FCT En or FCT BiH. No significant association was observed when comparing infants with FCT En to infants with FCT BiH or FCT Bi. We investigated this association further by stratifying the formula-fed infants by FCTs at 3 months and asking whether they differ with respect to time to first antibiotic use (Fig. 8). We observed that infants with FCT Bi microbiota had a 2 times higher hazard ratio (likelihood) to use antibiotics during the first year of life than the formula-fed infants with FCT BiH microbiota (delta = 2.077 [95% CI, 1.103 to 3.908]; *P* = 0.02). We did not observe any significant association between other reported clinical parameters and the 3-month FCTs.

## DISCUSSION

In this study, we characterized the effect of supplementation of an infant formula with two HMOs on the microbiota of healthy infants during the intervention and its persistence after cessation of the 6-month intervention (samples at 3 and 12 months, respectively).

Using a classical clades-based analytical approach, the microbiota of formula-fed 3-month-old infants was different if they received HMOs and closer to the microbiota of BF infants. This was observed for the microbial diversity, the global composition at the genus level, and the abundance of several major genera typical of that age period. Among these taxa, we observed an increase of bifidobacteria, which are believed to exert positive health benefits on their host (34). We also observed a decrease of *Escherichia* and unclassified *Peptostreptococcaceae*, a family to which Clostridium difficile belongs (35). This is potentially a beneficial effect of the two-HMO supplementation, considering that a high abundance of Escherichia coli or C. difficile has been associated with the development of eczema or atopy, respectively (36).

In general, the Caesarean-delivered infants showed more differences between formula groups than the vaginally delivered infants. Since the early gut microbiota of infants is altered by Cesarean compared to that by vaginal delivery (5, 6, 9), these results suggest a stronger normalization effect of the two HMOs on altered or dysbiotic microbiota. Similarly, breastfeeding was previously shown to better correct the dysbiotic gut microbiota of C-section delivered infants (9), especially when containing 2′-fucosyl-HMOs such as 2′FL (37).

At 12 months, the microbiota profiles changed from those observed at 3 months and no longer significantly differed between the formula groups, although several trends may persist. On the other hand, the BF group still significantly differed from the formula groups, but to a lesser extent.

The strategy that we used to annotate the short 16S rRNA reads (15) accurately distinguishes between most of the species and subspecies of bifidobacteria commonly found in infants. Therefore, we were able to establish by 16S rRNA sequencing that the distributions of the different *Bifidobacterium* species and subspecies abundances were not significantly different between the feeding groups at 3 months of age. Notably, and despite high levels of bifidobacteria in most 3-month samples, B. longum subsp. *infantis* was, in most infants, a minor component of the stool microbiota in our study. This subspecies showed no significant difference of distribution between feeding groups and, therefore, was not the most favored bifidobacterial species in the presence of HMOs, contrary to what was previously suggested (38). Similarly, the association between the secretory status of mothers (affecting the abundance of 2′-fucosylated HMOs) and the abundance of B. longum subsp*. infantis* in breastfed infants, as originally observed in a U.S. cohort (39), could not be replicated in a study performed in Armenia and Georgia (40). Adding to our results obtained in two European countries, this observation suggests geography-related specificities of the infant gut bifidobacterial population in its ability to utilize HMOs. Recent literature indeed showed the metabolic capacity of strains from various bifidobacterial species (e.g., B. breve, B. longum subsp. *longum*, or *B. pseudocatenulatum*) efficiently utilizing HMOs (41–43). Noteworthy, the potential health benefits provided by distinct bifidobacterial species (and strains) remain to be established.

Using a modeling approach on the 3- and 12-month infant gut microbiota data set, we defined 7 clusters of samples showing similar taxon compositions at the genus level, dubbed fecal community types (FCTs). The distribution of these FCTs matched the sampling age, with three FCTs found exclusively at 3 months and four other FCTs essentially at 12 months.

At 3 months, approximately 20% of infants harbored an FCT En microbiota, whose frequencies were not different between feeding groups. In the succession of microbiota communities in infants, this *Enterobacteriaceae*-dominated community likely takes place before the bifidobacterium-dominated profiles (5, 41). Therefore, at 3 months, it may represent a small proportion of infants with a less mature microbiota. Both FCT BiH and FCT Bi were dominated by bifidobacteria. However, the FCT BiH, which was found in most BF infants and significantly more in the test than in the control group, showed a lower proportion of other bacteria than the FCT Bi. The latter FCT was significantly associated with the control formula group, while the proportion of infants with FCT BiH increased in the test formula group. FCT BiH and FCT Bi, with 92% and 76% of bifidobacteria, respectively, look very similar to the breastfed infant gut microbiota clusters B1 and B2 previously identified at 1 month using a completely different approach (41). In that study, they defined cluster E by its high level of *Enterobacteriaceae*, similarly to our FCT En. Then, they subdivided the *Bifidobacteriaceae*-dominated cluster B into clusters B1 and B2 based on the dominance of fucosyllactose-utilizing *Bifidobacterium* strains (as defined by the presence of a corresponding genetic factor in isolates). The coexistence of FCT BiH and FCT Bi in our test group fed the HMO formula may be linked to similar mechanisms.

Interestingly, we defined the FCTs at the genus level, similarly to the adult enterotypes previously reported (44). At 12 months, the FCTs seem to evolve toward the adult enterotypes: FCT Ba toward enterotype 1 (*Bacteroides*), FCT Pr toward enterotype 2 (*Prevotella*), and FCT Fi toward the more controversial (45, 46) and less defined enterotype 3 (*Ruminococcus/Blautia/Lachnospiraceae*). Although not as complex as the adult microbiota, these precursors indicate that the enterotype establishment occurs as early as 12 months for most infants, earlier than previously observed (47). Microbial communities in infant stool at 12 months were recently associated with cognitive development at 2 years of age (48). Using a different methodology (partitioning around medoids instead of DMM), three clusters were obtained showing little similarity with our FCTs and only “modest support” based on statistical scoring (as stated by the authors). In our study, FCT In seems to correspond to none of the known adult enterotypes and seems to represent an intermediate situation between the 3-month FCTs and the other 12-month FCTs. Recently, a study showed that the mothers’ secretor status affects the microbiota of their 2- to 3-year-old children (49). In children exclusively breastfed for at least 4 months of life, bifidobacteria were increased if the mothers were secretor positive, meaning they express functional fucosyltransferase 2 responsible for the synthesis of 2′-flucosyl-HMOs such as 2′FL in breastmilk. Similarly, FCT In (the highest in bifidobacteria at 12 months) was likely favored by our 6-month intervention with two HMOs, including 2′FL.

As recently observed in adult gut microbiota (50), the infant FCTs showed marked differences in total bacterial abundances. The higher relative abundance of bifidobacteria in FCT BiH was accompanied and amplified by a higher microbial load. Recently, it was shown that the bacterial cell amounts were decreased in the 6-month feces of infants with atopic dermatitis (51). In a follow-up study of Estonian and Swedish children who were sampled over the first year of life, a consistent lower level of *Bifidobacterium* in stools was associated with the development of allergy (52). These observations suggest a lower risk of allergy for infants carrying a FCT BiH. In our study, FCTs at 3 months associated prospectively with any antibiotic treatment in the first year of life. Infants showing at 3 months a stool microbiota of the type characterized by the highest abundance of bifidobacteria and the highest density of bacteria per gram of feces (FCT BiH) were less likely to require antibiotics up to 12 months. This also translated into a shorter time to first antibiotics use for infants with FCT Bi than with FCT BiH. Considering that antibiotics require physician prescriptions in Europe, the measure of their usage is a surrogate marker of infections. Therefore, this association is consistent with the observation that bifidobacteria confer protection against enteropathogenic infections through the production of acetate (53). The gut-lung axis is gaining credibility in the literature (54), with the gut microbiota impacting respiratory disease through the modulation of the immune response by short-chain fatty acids (SCFAs) (55, 56). Considering that the central metabolic pathway of bifidobacteria yields lactate and acetate as primary products of carbohydrate fermentation and that colonic acetate is efficiently absorbed (57), the modulation of the gut microbiota observed in our study may therefore explain the reported associations of two-HMO supplementation in infant formula with lower respiratory tract infection and medication use (30).

Our study shows some limitations, essentially depending on the original clinical trial powered for safety (30). Only two visits (3 and 12 months) were used for the collection of fecal samples, but the choice of the time points was informed to lower the burden for the parents, the risk of slowing down the recruitment, and the risk of increasing the dropout rate. Sampling stools at 3 months of age guaranteed that the infants (inclusion to study <14 days of age) were in the randomized feeding groups sufficiently long to see dietary effects on the microbiota, while not being too close to the time when feeding of complementary food (weaning) was allowed (4 months of age), as recommended by the pediatric societies. The 12-month stool sampling was suitable to explore any long-lasting effect of early-life HMO feeding. Missing data from the PP population (Fig. 1) decreased the power of our analyses but were not expected to bias the results, since they were the consequence of random processes: absence of sample collection (more pronounced for the 12-month samples) and insufficient number of sequences due to unequal pooling of PCR products before the multiplexed sequencing. Presence of older siblings, introduction of solid food (weaning), and daycare attendance are major determinants of exposure to infectious agents that might result in antibiotic treatment. Even though the percentage of siblings was not different between the treatment groups, the precise timing of the introduction of solid food (allowed after 4 months of age) and daycare attendance were not available for assessment and may have introduced biases. However, randomization of the infants at recruitment should have removed possible differences between groups. Using samples from a safety clinical trial, the current analysis of microbiota is an exploratory work to formulate hypotheses for future research and to inform future designs of clinical trials.

Overall, the addition of two individual structurally very specific HMOs to a starter infant formula shifts the microbiota toward the microbiota observed with breastfeeding, the standard in infant nutrition. This suggests that the risks for diseases linked to gut ecology may be shifted toward the reduced risk level generally observed in breastfed infants (58, 59). In our study, the association of the two-HMO-promoted FCT BiH with an infection-related marker of positive health outcomes (less antibiotics usage) provides arguments for this paradigm.

## MATERIALS AND METHODS

### Trial design and participants.

The trial was conducted from October 2012 through July 2015 at the Dipartimento Materno Infantile AOUP Paolo Giaccone Università di Palermo in Palermo, Italy, and the Department of Paediatrics at Jessa Hospital in Hasselt, Belgium. Trial conduct complied with the Declaration of Helsinki and the International Conference on Harmonization guidelines for good clinical practice. Prior to enrollment, informed consent was obtained from the parent or the legal representative of the infants. The study was approved by the Ethical Committees of Jessa Hospital (Belgium) and the University of Palermo (Italy).

Healthy full-term (37 weeks ≤ gestational age ≤ 42 weeks) infants with a birth body weight between 2,500 g and 4,500 g, younger than 14 days, and exclusively formula fed at enrollment were eligible. Before enrollment, mothers independently elected not to breastfeed. In the breastfed reference group, infants exclusively breastfed since birth and whose mothers intended to exclusively breastfeed at least to 4 months were screened for enrollment at 3 months (±5 days) of age. The target was to enroll 40 exclusively breastfed infants stratified to have equal numbers by sex and study site. Exclusion criteria included (i) congenital illness or malformation that may affect growth, (ii) significant prenatal and/or serious postnatal disease before enrollment (by medical decision), (iii) minor parent(s), (iv) newborn whose parents/caregivers cannot be expected to comply with study procedures, and (v) concurrent participation or prior participation in another clinical trial since birth, except for BF group, where vaccine studies were allowed. Infants were randomly assigned to one of two study formulas using mode of delivery (vaginal or Caesarean) and sex as stratification factors. Randomization was carried out using a permuted block algorithm with Medidata Balance (New York, NY, USA). Randomized infants received exclusive feedings with the test or control formulas from enrollment through 4 months of age, in amounts suitable for their weight, age, and appetite. Introduction of weaning (solid) food was allowed from 4 months, with continuation of the control or test formula until 6 months of age. Then, both groups received the same intact protein cow’s milk-based follow-up formula until 12 months of age. Parents/caregivers, investigators, and study support staff were blinded to the identity of the study formulas. The study formulas were coded by the Nestlé Product Technology Center (Konolfingen, Switzerland) with nonspeaking codes. The study flow chart is depicted in Fig. 1.

### Interventions.

The control formula was an intact protein cow’s milk-based whey-predominant infant formula with long-chain polyunsaturated fatty acids (LC-PUFA; 66.9 kcal/100 ml reconstituted formula, 1.889 g protein/100 kcal powder with a whey:casein ratio of 71.6:28.4). The test formula was the same recipe and ingredients as the control formula, supplemented with 2 HMOs (2′-fucosyllactose and lacto-N-*neo*tetraose) at target minimum and maximum concentrations of 1.0 to 1.2 g/liter of reconstituted formula for 2′FL and 0.5 to 0.6 g/liter of reconstituted formula for LNnT, replacing an equivalent amount of lactose.

### Clinical parameters.

The measured parameters included antibiotics use, antipyretics use, gastro-esophageal reflux disease (GORD) treatments, system organ class (SOC) gastrointestinal disorder, SOC respiratory disorder, SOC infection, preferred-term (PT) bronchiolitis, PT bronchitis, PT gastrointestinal disorder, PT rhinitis, PT upper respiratory tract infection, PT viral respiratory tract infection, adverse events (AE) cluster grouping for lower respiratory tract illnesses, and otitis, cumulatively assessed at 4, 6, and 12 months (the detailed list of variables is described elsewhere [30]).

### Stool collection.

Within 48 h preceding the 3- and 12-month visits, stool samples were collected by parents and stored at home in their −20°C freezer. To this end, parents were supplied a kit (insulated bag, ice pack, spatula pots, sealable plastic bags, and instruction sheet). Stool samples were transported within insulated bags containing an ice pack to the site of the visit where they were kept frozen at −80°C. Samples were then shipped to the Nestlé Research Center, Switzerland, on dry ice and kept frozen at −80°C until analysis.

### Fecal DNA extraction and microbiota composition analysis.

Total DNA was extracted from fecal samples using the QIAamp DNA Stool minikit (Qiagen) according to the manufacturer’s instructions, except for the addition of a series of mechanical disruption steps (4 × 60 s) using a FastPrep apparatus and Lysing Matrix B tubes (MP Biochemicals). Notably, this protocol is very similar to the protocol recommended by the International Human Microbiome Standards (60). Microbiota composition was analyzed by sequencing of the V3 and V4 regions of the 16S rRNA gene using a recently described approach (15). In short (see Fig. S4 in the supplemental material), paired-end sequences were assembled into contigs and quality filtered using Mothur (61). Then, chimera filtering was performed using QIIME (62). After a taxonomic annotation at the genus level of each contig using Mothur, sequences belonging to the genera *Bifidobacterium* and *Lactobacillus* were extracted and annotated to the species or subspecies level based on signature sequences of the 16S rRNA genes (15). The other contigs were clustered into OTUs and annotated using QIIME. All sequences were combined into one final OTU table using QIIME’s open reference approach. After quality filtering of the OTUs (63), diversity analyses were performed using QIIME and the website Calypso (64) at http://cgenome.net/calypso. Dirichlet multinomial mixture analysis was performed using Mothur. The optimal number of clusters to describe our data set was assessed 10 times using the Laplace approximation to the negative log model evidence. The clustering with the lowest Laplace value was selected. Taxon abundance comparisons between feeding groups at the genus level were performed with nonparametric Mann-Whitney U tests or Kruskal-Wallis tests and controlled for the error rate (65). Only results with uncorrected *P* values of <0.05, false-discovery rates of <0.25, and for taxa showing medians of more than 0.1% in the formula groups were reported. GraphPad Prism version 6.07 for Windows (GraphPad Software, La Jolla California USA) was used for data in some figures.

### Statistical analysis of the fecal community types distributions.

For the analysis of microbiota FCTs, descriptive statistics was performed by cross tabulation of FCT and feeding groups. The treatment differences of each FCT between two feeding groups were analyzed by χ^2^ tests. The *P* values were derived from an approximated null distribution estimated by bootstrapping (independence_test library [coin]). This method is expected to give reliable *P* values even when zero cell counts are present. The time to first antibiotic use was analyzed by a Cox-proportional hazard model with explanatory variable FCTs at 3 months.

10.1128/mBio.03196-19.4 FIG S4 Overview of the 16S rRNA analytic pipeline previously described in reference 15. Colors refer to the software used for the different steps, commands in brackets refer to specific scripts and options that were executed. *, in the present paper, the sequences annotated as *Streptococcus* were processed with the other contigs and without the use of custom scripts. Download FIG S4, PDF file, 0.4 MB. Copyright © 2020 Berger et al. 2020 Berger et al. This content is distributed under the terms of the Creative Commons Attribution 4.0 International license.

### Quantification of total 16S rRNA gene load.

The total number of 16S rRNA gene copies was determined per gram of feces as previously described (66). Distributions in feeding groups or FCTs were compared by Kruskal-Wallis and Dunn’s multiple-comparison tests.

### Data availability.

16S rRNA gene sequencing raw data were deposited in SRA under accession number SRP151522.
