# Supplementary figures and images for: Linking Human Milk Oligosaccharides, Infant Fecal Community Types, and Later Risk To Require Antibiotics
Source: mBio. 2020 Mar 17;11(2):e03196-19. doi: 10.1128/mBio.03196-19 (PMC7078481; doi:10.1128/mBio.03196-19)

3m

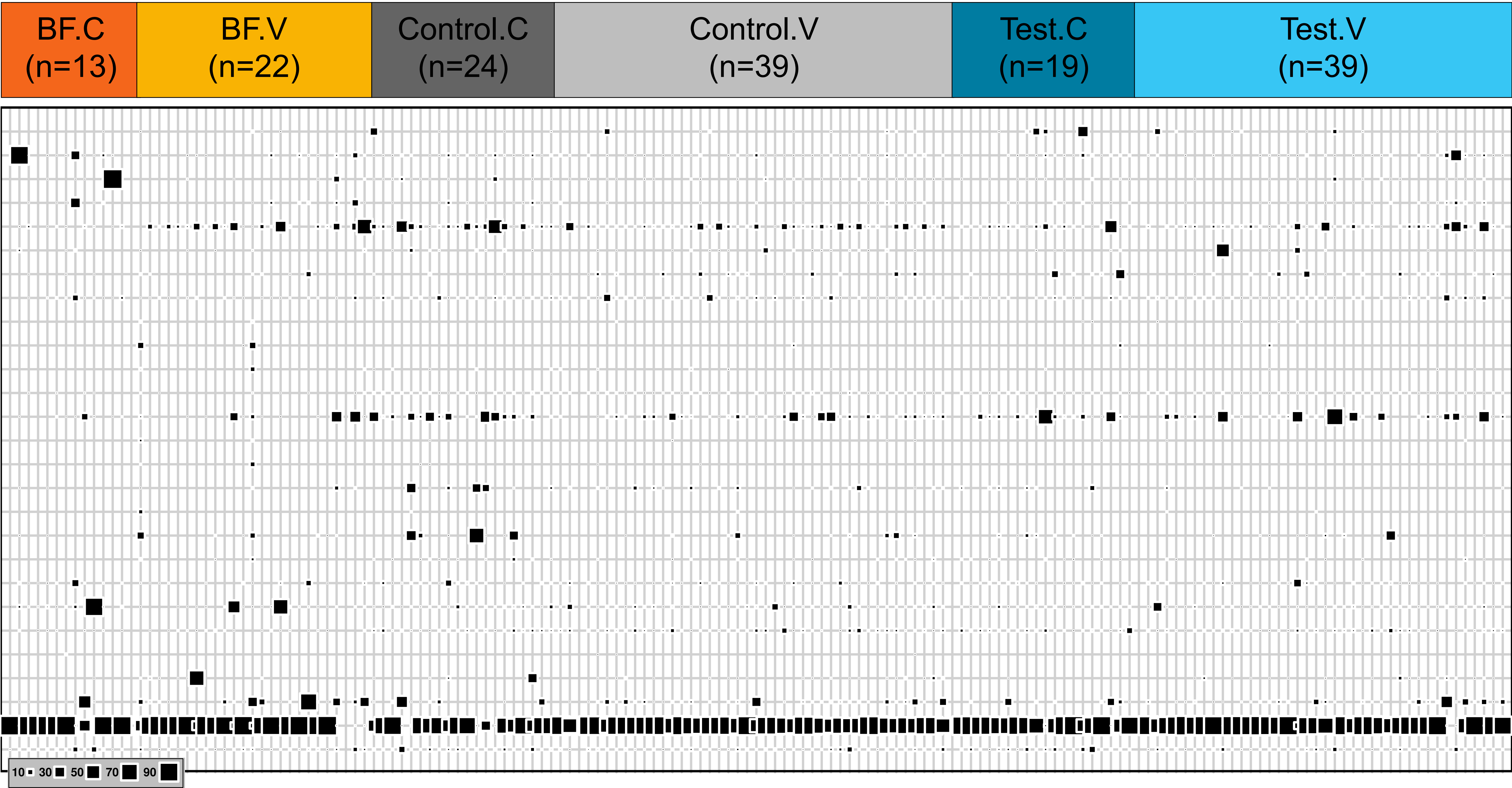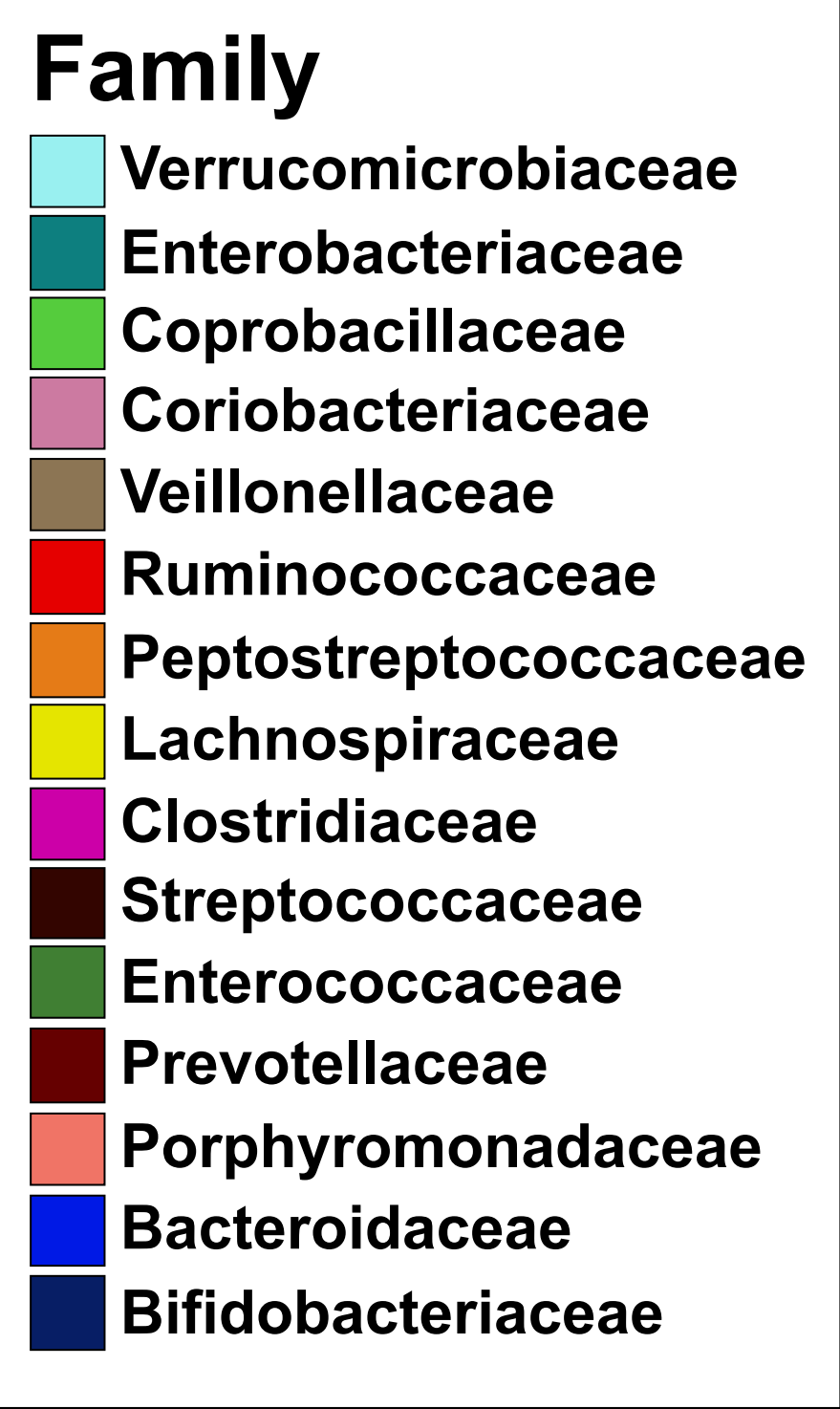

12m

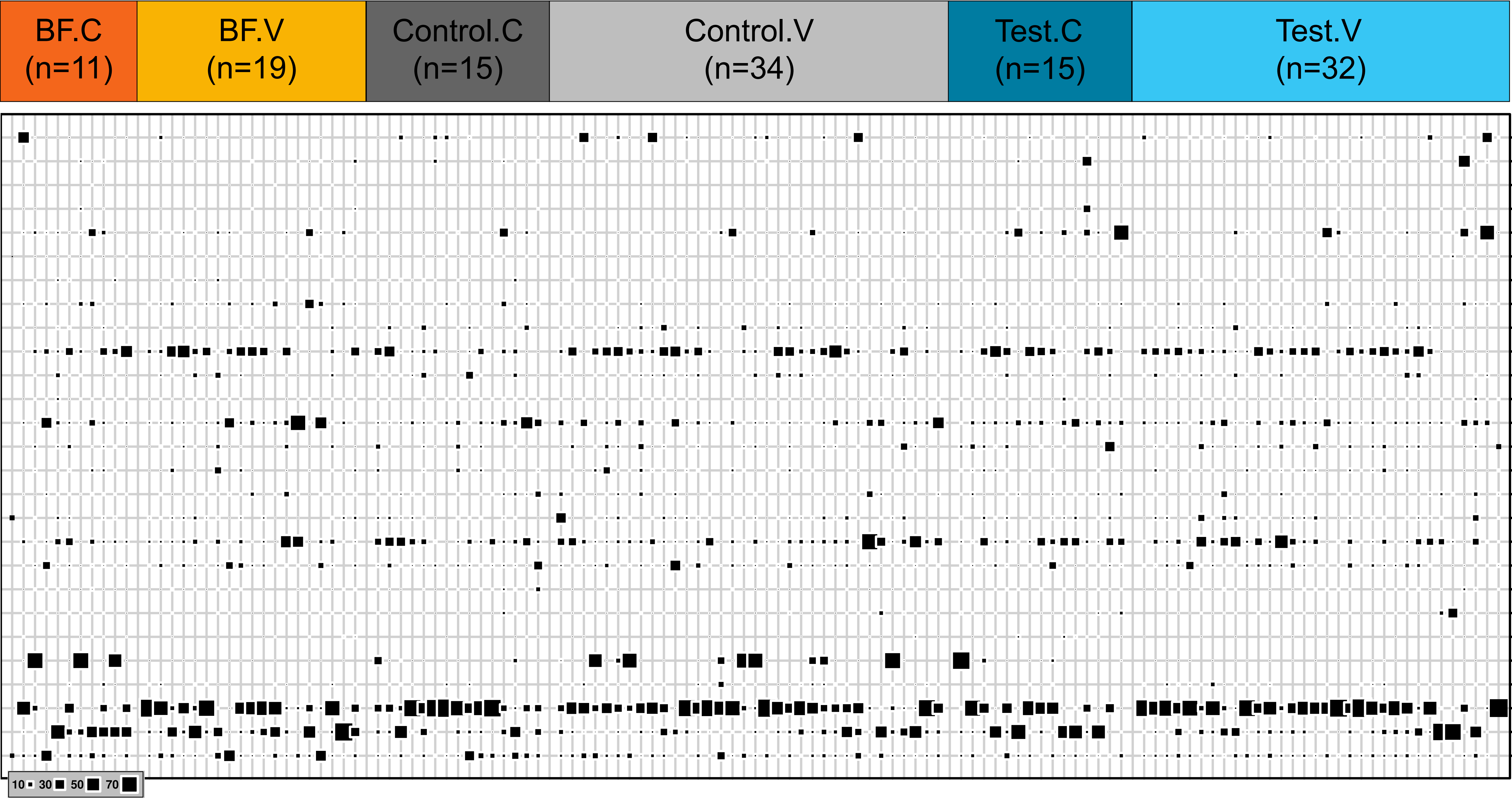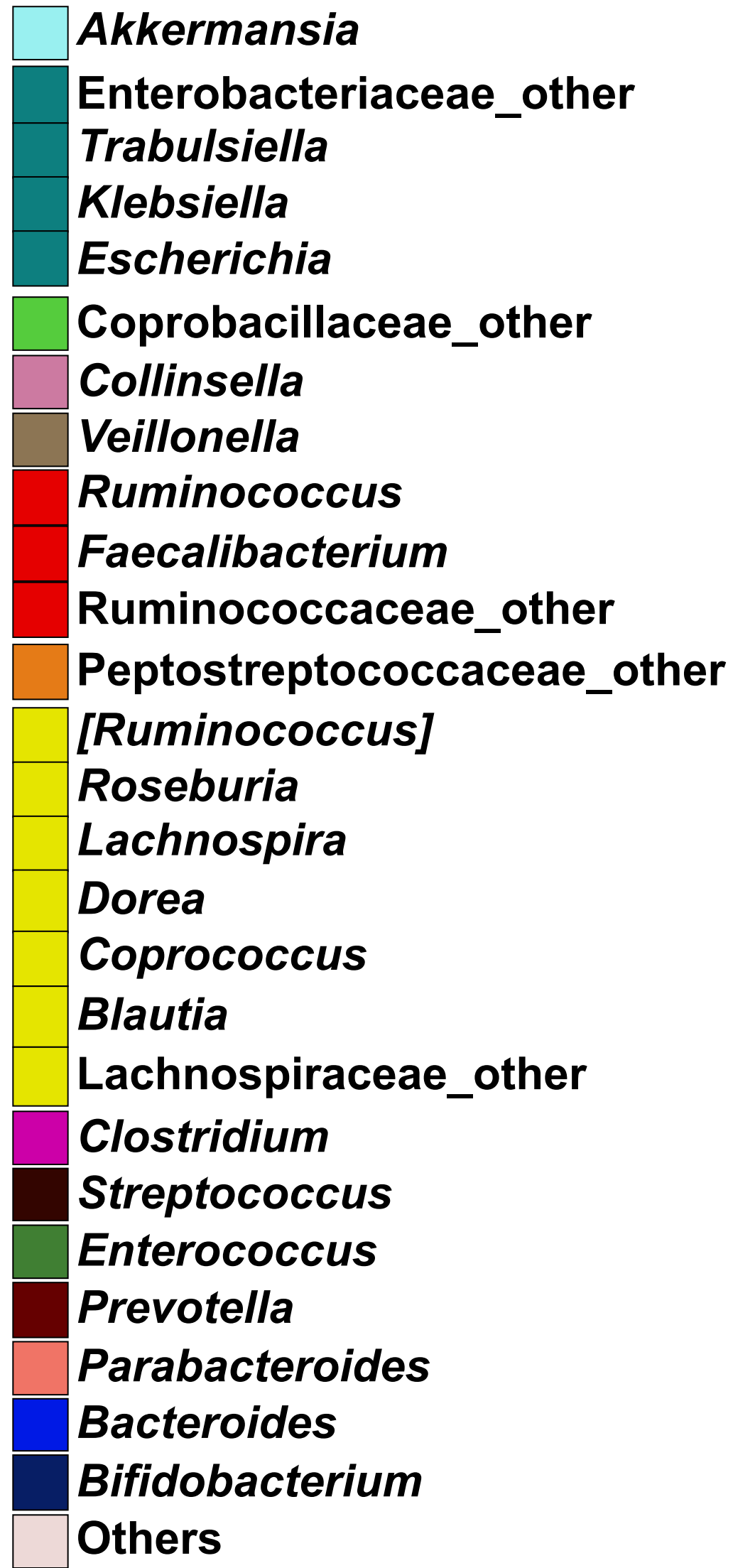

Supplement: FIG S1 [file mBio.03196-19-sf001.pdf]

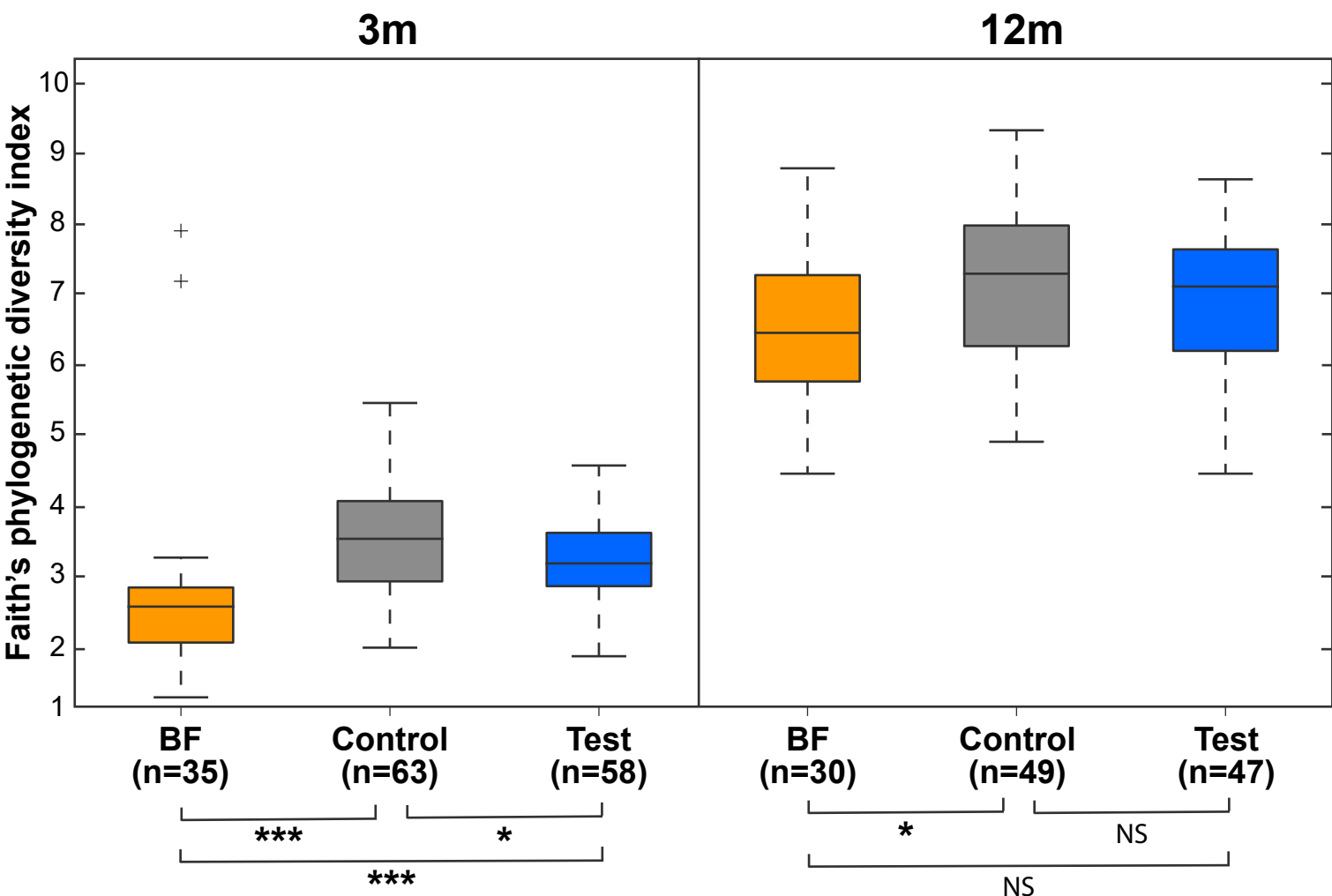

Supplement: FIG S2 [file mBio.03196-19-sf002.pdf]

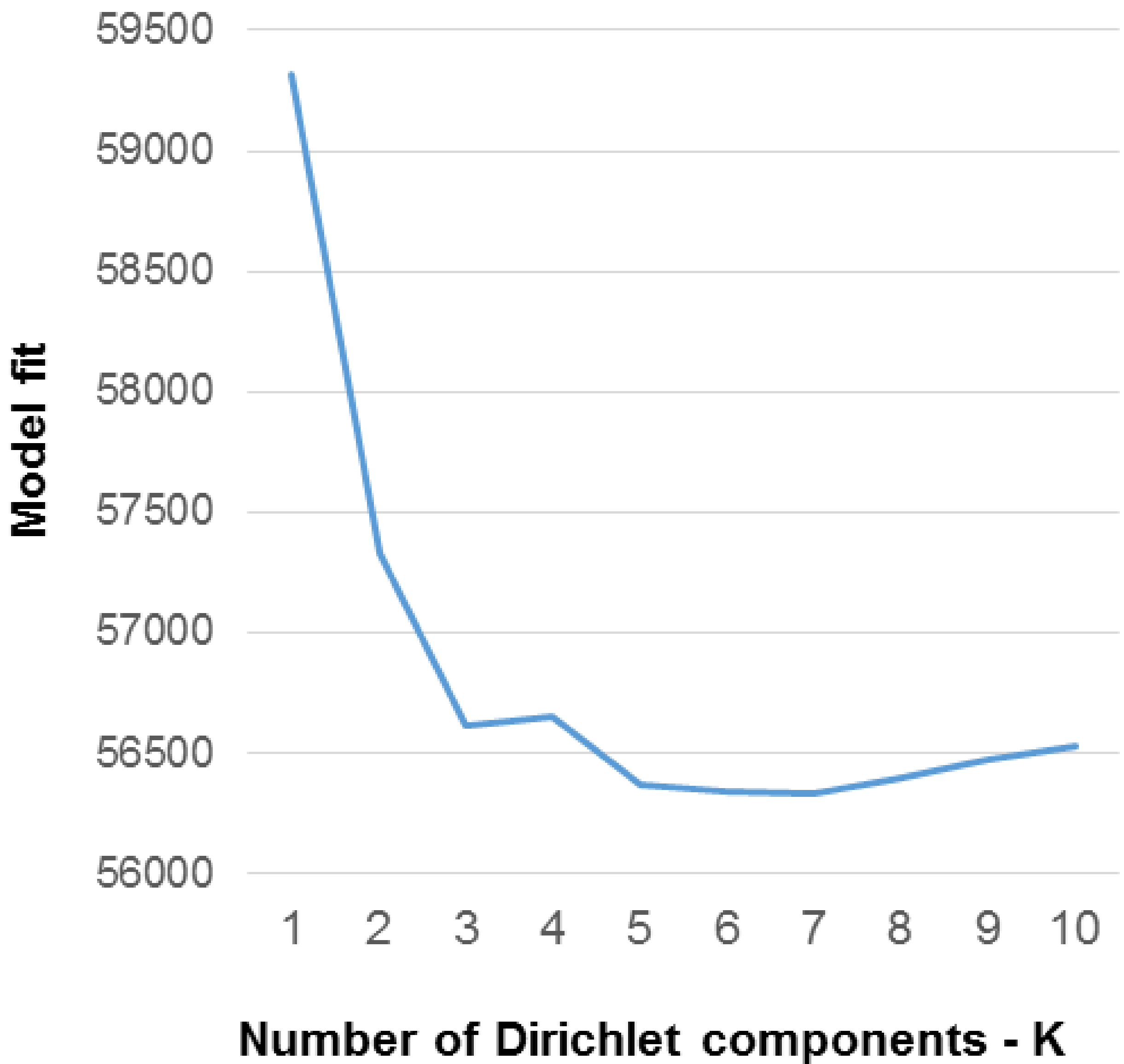

Supplement: FIG S3 [file mBio.03196-19-sf003.pdf]

# 16S rRNA analytic pipeline

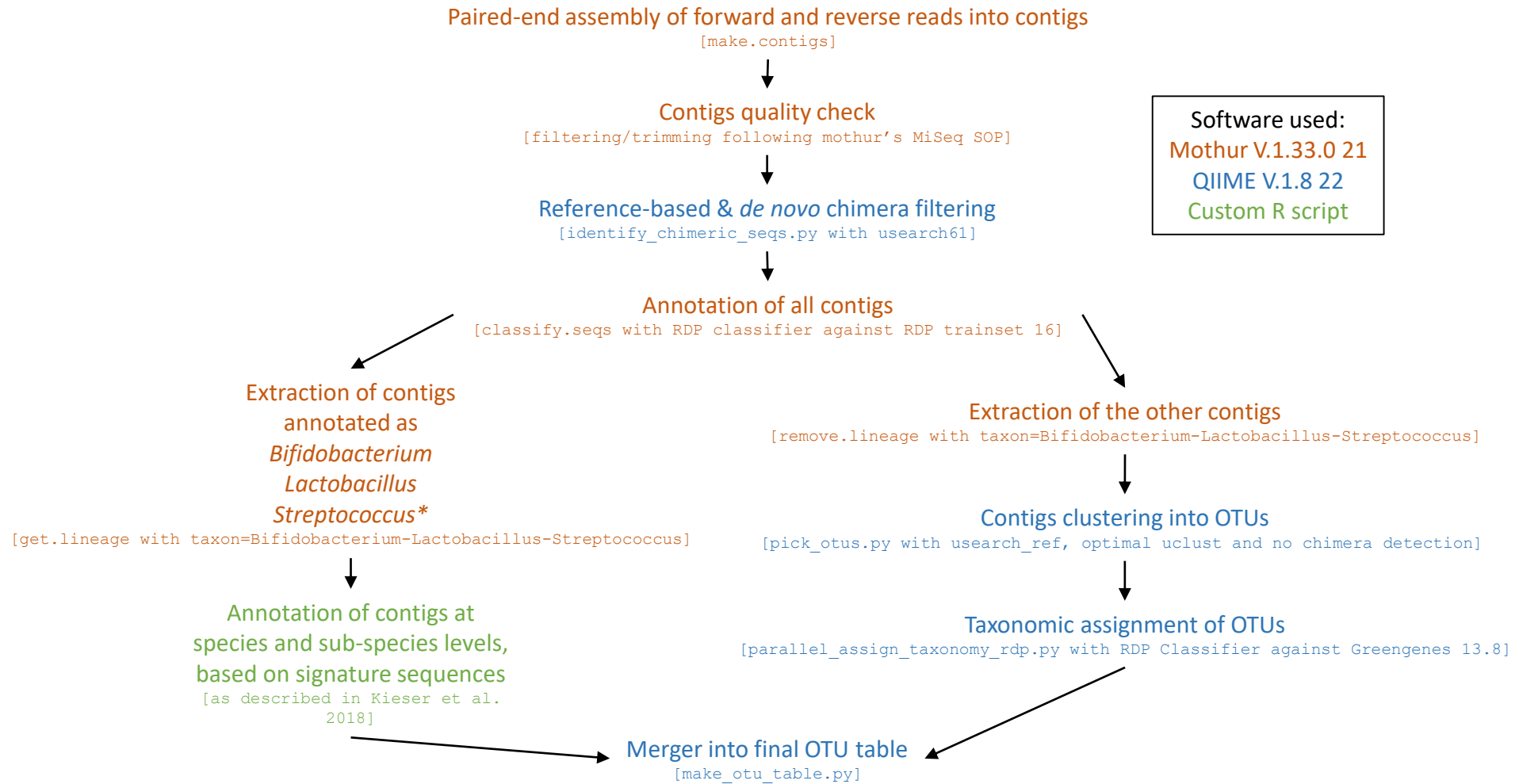

Supplement: FIG S4 [file mBio.03196-19-sf004.pdf]
