# Supplementary material for: Linking Human Milk Oligosaccharides, Infant Fecal Community Types, and Later Risk To Require Antibiotics
Source: mBio. 2020 Mar 17;11(2):e03196-19. doi: 10.1128/mBio.03196-19 (PMC7078481; doi:10.1128/mBio.03196-19)
Supplement: TABLE S1 [file mBio.03196-19-st001.pdf]

Supplementary Table 1. *Separation between feeding groups and delivery modes at 3 months assessed by the distribution Bray-Curtis distances at genus level*

	within group 1		within group 2		between groups		
Comparison group 1 vs. group 2	average	variance	average	variance	average	variance	p-value*
Test.Vaginal vs. Test.Caesarean	0.39	0.11	0.75	0.081	0.39	0.094	0.21
Control.Vaginal vs. Control.Caesarean	0.26	0.011	0.57	0.079	0.49	0.081	2.5×10^{-16}
BF.Vaginal vs. BF.Caesarean	0.5	0.15	0.61	0.19	0.55	0.18	0.12
Test.Caesarean vs. Control.Caesarean	0.39	0.081	0.57	0.079	0.51	0.09	0.31
Test.Vaginal vs. Control.Vaginal	0.39	0.11	0.26	0.011	0.33	0.063	0.11
BF.Vaginal vs. Test.Vaginal	0.5	0.15	0.39	0.11	0.45	0.13	0.076
BF.Vaginal vs. Control.Vaginal	0.5	0.15	0.26	0.011	0.41	0.088	6.4×10^{-8}
BF.Caesarean vs. Test.Caesarean	0.61	0.19	0.39	0.081	0.53	0.14	0.016
BF.Caesarean vs. Control.Caesarean	0.61	0.19	0.57	0.079	0.64	0.11	6.4×10^{-5}

*P-value of the Wilcoxon rank test of the hypothesis “within groups distances < between groups distances”
