# Supplementary material for: Linking Human Milk Oligosaccharides, Infant Fecal Community Types, and Later Risk To Require Antibiotics
Source: mBio. 2020 Mar 17;11(2):e03196-19. doi: 10.1128/mBio.03196-19 (PMC7078481; doi:10.1128/mBio.03196-19)
Supplement: TABLE S2 [file mBio.03196-19-st002.pdf]

Supplementary Table 2. *Differences of taxa abundances at genus level between Control and Test groups at 3 months.*

At 3 months	Statistics (Test/Control)		Median			Mean		
Taxa	p-value	FDR	Control	Test	BF	Control	Test	BF
Escherichia	0.0078	0.13	4.42	1.56	1.82	8.13	4.79	4.54
Bifidobacterium	0.0093	0.13	74.47	82.81	90.87	64.75	70.40	64.49
Peptostreptococcaceae_*g__unassigned*	0.0275	0.16	0.31	0.18	0.00	0.44	0.26	0.03
Streptococcus	0.0372	0.17	0.46	0.27	0.11	1.46	1.00	5.95

Values for the BF group are shown as references. Values for Test, Control and BF are medians and means of the relative abundance of the indicated genus (% of all sequences). P-value : Mann-Whitney U tests of the Test and Control groups. FDR : false discovery rate
