# Supplementary material for: Linking Human Milk Oligosaccharides, Infant Fecal Community Types, and Later Risk To Require Antibiotics
Source: mBio. 2020 Mar 17;11(2):e03196-19. doi: 10.1128/mBio.03196-19 (PMC7078481; doi:10.1128/mBio.03196-19)
Supplement: TABLE S3 [file mBio.03196-19-st003.pdf]

Supplementary Table 3 *Differences of abundances for bifidobacterial species between BF, Control and Test groups at 3 months.*

At 3 months	Statistics	Median			Mean			
Taxa	p-value	Control	Test	BF	Control	Test	BF	All
Bifidobacterium_s__adolescentis.S	0.24	0.01	0.01	0.00	1.54	2.86	0.22	1.73
Bifidobacterium_s__animalis.subsp.lactis.S	0.55	0.00	0.00	0.00	2.06	0.02	0.00	0.84
Bifidobacterium_s__bifidum.S	0.79	2.42	7.57	6.01	10.29	9.44	17.47	11.58
Bifidobacterium_s__breve.S	0.72	0.33	1.19	0.11	11.32	17.76	17.61	15.13
Bifidobacterium_s__catenulatum.pseudocatenulatum angulatum.kashiwanohense.S	0.6	0.03	0.05	0.03	13.19	12.23	3.98	10.77
Bifidobacterium_s__dentium.S	0.66	0.01	0.01	0.01	1.19	1.57	0.27	1.12
Bifidobacterium_s__longum.subsp.infantis.suis.S	0.89	0.03	0.04	0.04	3.59	4.82	9.20	5.31
Bifidobacterium_s__longum.subsp.longum.S	0.74	3.41	11.63	2.61	19.73	19.27	13.83	18.24
Bifidobacterium_s__scardovi.S	0.14	0.00	0.00	0.00	0.12	0.31	0.01	0.17
Bifidobacterium_Other	0.22	0.64	1.70	0.33	1.34	1.50	1.39	1.41

Values for Test, Control and BF are medians and means of the relative abundance of the indicated taxa. P-value : Kruskal-Wallis test of the three groups.
